# Exploring sex as a moderator of other prognostic variables in whiplash associated disorder: An observational study

**DOI:** 10.1371/journal.pone.0282640

**Published:** 2023-04-26

**Authors:** Bradford Callan, David M. Walton, Joshua Cleland, Morey J. Kolber, James M. Elliott

**Affiliations:** 1 School of Physical Therapy, Pacific Northwest University, Yakima, Washington, United States of America; 2 Physical Therapy and Health and Rehabilitation Science, Western University, London, Ontario, Canada; 3 Doctor of Physical Therapy Program, Tufts University School of Medicine, Boston, Massachusetts, United States of America; 4 Doctor of Physical Therapy Program, Nova Southeastern University, Fort Lauderdale, Florida, United States of America; 5 The Kolling Institute of Medical Research, The University of Sydney and The Northern Sydney Local Health District, St. Leonards, New South Wales, Australia; Complutense University of Madrid: Universidad Complutense de Madrid, SPAIN

## Abstract

**Background:**

Prognostic variables for assessing people with whiplash associated disorder (WAD) following a motor vehicle collision (MVC) have been evaluated in numerous studies. However, there is minimal evidence assessing how these variables may differ between males and females.

**Question/Purpose:**

1) To assess if the sex of a person interacts with known prognostic variables within the development of chronic WAD. 2) To determine if commonly used outcome measures used in the assessment of chronic WAD differ between sexes.

**Methods:**

The study was a secondary analysis of an observational study with an inception cohort immediately following an MVC in an emergency department in Chicago, IL, USA. Ninety-seven adults aged 18 to 60 (mean 34.7 years old; 74% female) participated in the study. The primary outcome was long-term disability as determined by Neck Disability Index (NDI) scores at 52-weeks post-MVC. Data was collected at baseline (less than 1-week), 2, 12, 52-weeks post MVC. Hierarchal linear regression was used to determine significance (ΔF-score, p < 0.05) and R^2^ for each of the variables. The primary variables of interest were sex of the participant, age, baseline scores on the numeric pain rating scale (NPRS) and NDI and created interaction terms for sex *x* z-baseline NPRS and sex *x* z-NDI.

**Results:**

From analysis 1, both NDI (R^2^ = 8.7%, p < 0.01) and NPRS (R^2^ = 5.7%, p = 0.02) collected at baseline predicted significant variance in NDI score at 52-weeks. The interaction term of sex x z-NPRS was also significant (R^2^ = 3.8%, p = 0.04). In analysis 2 the regression models when disaggregated by sex showed that baseline NDI was the significant predictor of 52-week outcome in males (R^2^ = 22.4%, p = 0.02) while it was the NPRS as the significant predictor in females (R^2^ = 10.5%, p < 0.01).

## Introduction

Non-catastrophic motor vehicle collisions (MVC) (those that do not result in fractures, loss of limbs, or death) are common in the United States with over 6 million reported each year [1]. It is estimated over one-million people will present immediately to the emergency department (ED), or to their physician for sustained injuries, with the most common being ‘whiplash-associated disorder’ (WAD) [1, 2]. Signs and symptoms of WAD include, but are not limited to: neck or widespread pain, weakness, psychological distress, tinnitus, headache, swallowing deficits, balance, and oculomotor deficits [3]. Longitudinal modeling studies indicate that about 50% of those with WAD will fully recover in the first 6 to 12-weeks, while the remaining 50% will continue to report persistent symptoms in the long-term [3, 4]. There remains no gold standard diagnostic test for WAD. Rather, it is generally diagnosed by the presence of neck pain, stiffness, or tenderness only (no physical signs–Quebec Task Force (QTF) grade I); neck pain and stiffness (QTF grade II); neck pain and peripheral neurological signs (QTF grade III); and neck pain and fracture or dislocation (QTF grade IV) following an MVC or other mechanical event (e.g., sports injury, slips, and falls) through which energy is rapidly transmitted between the trunk and head through the neck [5].

Recovery from WAD is usually recorded using patient self-reported outcome measures (PROMs). Two of the most common PROMs are the Neck Disability Index (NDI) [6] and the 0–10 Numeric Pain Rating Scale (NPRS) [4]. The NDI is a 10-item scale intended to capture self-ratings of disability associated with neck pain [6]. It is one of the most widely used neck-specific disability scales, with a recognizable scoring system that is commonly reported between 0% (no disability) and 100% (complete disability) [7]. The NPRS is a single-item tool that is used to estimate the intensity of pain a person is experiencing and is amongst the most ubiquitous PROMs used globally [8]. Two prior latent growth curve analyses have found that PROM-based recovery from acute WAD can be defined through a 3-trajectory model: a rapid/full recovery group, a delayed recovery/mild disability group, and a non-recovery/severe disability group [3, 9] with proportions roughly matching the 50% recovered and 50% not recovered estimates. Subsequently, in the interest of identifying more homogenous subgroups and more informed early management decisions to prevent persistent problems, considerable prognostic work has been conducted to help clinicians and researchers better estimate the most likely recovery outcomes of people with acute WAD. Through this body of work, baseline (acute) pain severity (NPRS) and disability scores (NDI) have been consistently endorsed as strong predictors of 12-month outcomes [4].

To date, the majority of prognostic work has not considered the potential for differential functioning of prognostic factors across clinically relevant subgroups. One of the most obvious would be the possibility that the accuracy of prognostic variables is moderated by the sex of the injured person. Some prior work in the field has identified sex itself as a potential prognostic variable, wherein females are more likely to report persistent pain than are males following WAD [10]. However, as a target for intervention, it is unclear how sex can or should considered to improve outcomes. Rather we are informed by the Sex and Gender Equity in Research (SAGER) initiative [11], endorsing exploration of research findings disaggregated by sex, whereby more equitable evidence for clinical decision making can be realized, rather than assuming variables function the same in both males and females.

While both high NDI and high NPRS scores have established evidence as risk factors for persistent pain and disability post-WAD, the purpose of this study was to follow the SAGER guidelines and explore the potential that the strength of that prognosis varies as a function of the respondent’s sex.

## Methods

### Participants

This study was a secondary analysis of data collected during a parent study investigating recovery from WAD (ClinicalTrials.gov Identifier: NCT02157038). In all, data were collected from 97 participants (72 females) who were recruited from an academic emergency department (ED) in Chicago, IL, USA from September 2014 through May 2019. To be eligible for the study, the following inclusion criteria had to be met: an MVC within the previous week with a QTF whiplash grade of 2 or 3 [5], and be between 18 to 65 years of age. Exclusion criteria were a previous MVC at any time, treatment for neck pain disorders in the past ten years, any nervous system disorder (e.g., stroke, Parkinson’s), metabolic system disorder (e.g., diabetes), or those who, by standard emergency medical services’ protocols were deemed to be at risk for multi-system trauma. The Institutional Review Board of Northwestern University Feinberg School of Medicine granted approval and all participants provided written informed consent (STU00090769-MODCR0003).

### Primary outcome measure

The primary recovery outcome of the parent study was NDI score, reported as 0–100% disability. The NDI has been extensively studied in people with traumatic and non-traumatic neck pain [12], and it has demonstrated adequate reliability and validity and has been deemed appropriate to use in both clinical and research settings based on its reported psychometric properties [13, 14]. NDI scores were captured at inception (≤1 week from injury) and again at 2, 12, and 52 weeks follow-up. For this study only inception and 52-week scores were extracted, ostensibly indicating where participants started and where they ended in the study. For those lost to follow-up (n = 19), the 12-week score was carried forward as prior modeling and reviews evaluating participants with WAD grades 2 and 3 has revealed very little change occurs between 12 and 52 weeks [3, 4].

### Predictor variables

The variables collected for this study were sex-at-birth (male/female), age in years, and scores on the NDI and NPRS at inception. The NPRS is an 11-point scale that ranges from 0 to 10 with scores on each whole integer [15]. The anchors for the scores are 0 = no pain and 10 = the worst pain imaginable. The psychometrics of the NPRS have been reported across various subgroups, including those with WAD, and it is reported to be an adequately valid, reliable, and responsive tool [13, 16].

### Statistical analysis

Participant characteristics (sex, age, baseline NDI and NPRS) were reported descriptively (proportions or means and range), see Table 1. Missing data were first assessed with Little’s Missing Completely At Random (MCAR) test, and then handled on a case-by-case basis. As a first pass analyses, we explored the potential for differences in mean NDI and NPRS scores at baseline when the sample was disaggregated by sex using independent t-tests or Mann-Whitney U-tests dependent on the structure of the data, see Table 1.

**Table 1:** Based on participant sex: Independent *t*-test for age and baseline NDI scores, and Mann-Whitney U test for baseline NPRS.

	Male (n = 25) Mean (95%CI)	Female (n = 72) Mean (95% CI)	*p*-value
**Age**	32.1 (28.0 to 36.3)	35.6 (32.9 to 38.2)	0.19
**NPRS at baseline**	5.2 (4.1 to 6.2)	5.0 (4.5 to 5.5)	0.88
**NDI at baseline**	32.2 (24.2 to 40.2)	37.6 (33.9 to 41.1)	0.17

Abbreviations: NPRS: numeric pain rating scale; NDI: neck disability index; CI: confidence interval.

For the primary analyses, we used multiple stepwise (hierarchical) linear regression. The dependent variable was NDI score at 52-weeks, and the potential predictor variables in the first analysis were (in order of entry): sex, age, NPRS, and NDI scores at baseline. In the second model we added two interaction terms after the same four variables: sex *x* z-NPRS and sex *x* z-NDI. We transformed the baseline NPRS and NDI scores to z-scores to reduce the risk of multicollinearity as both baseline NPRS and NDI scores were used in the first steps of the model.

For both models, variables were retained when their inclusion led to a significant (p<0.05) change in F-value, and the size of the effect was interpreted through the R^2^ as a percent of unique variance in 52-week NDI score explained by that variable. Where inclusion of the interaction terms was significant, we interpreted that as an indication the prognostic value of that variable was different between the sexes. For illustrative purposes and in accordance with SAGER guideline [11], when a significant interaction was identified we repeated the first regression model with the sample disaggregated by sex to enable reporting of estimated variance in 52-week NDI score explained by each variable for each sex.

Prior to evaluating the models, all assumptions regarding linear regression were assessed. Linearity was assessed by partial regression plots of all IV’s (except sex as it is categorical) against the DV 52-week NDI score, homoscedasticity was evaluated by plotting standardized residuals against the predicted residuals, multicollinearity was assessed by looking for correlation coefficients greater than 0.75 and tolerance less than 0.1, standardized residuals were assessed with a P-P plot, outliers were determined if any of the standardized residuals were greater than 3 standard deviations from a mean of 0, a leverage point was considered significant if it was greater than 0.2, and data were considered to be an influential point if Cook’s distance was greater than 1 [17].

An a priori sample size estimate was performed, and with an 80% power and alpha set at 0.05, assuming a 10% dropout rate, 100 participants, with an expected 10% drop-out rate (n of 90), were necessary to achieve the desired results within the original NIH study parameters. Secondary sex-disaggregated analyses were expected to be underpowered when the sample was split, so these were considered exploratory hypothesis-generating analyses only.

All analyses were performed using SPSS Statistics for Windows Version 26.0 (IBM Corporation, Armonk, NY).

## Results

### Participants

A total of 100 people consented to participate of which 97 (72 female) presented for their initial assessment. All 97 provided data at baseline, 2- and 12 weeks, while 78/97 provided data at all 4 timepoints. Of the 19 (19.6% of baseline) that were lost to follow up between 12 and 52 weeks, 5 were male and 14 were female. Proportions of males and females were not different between the baseline and 52-week collection periods (ꭓ^2^ = < 0.01, p = 0.95). MCAR test indicated a random pattern of missing data (ꭓ^2^ = 4.59, p = 0.47). Considering this, and prior evidence revealing no significant difference in mean NDI scores between 12 and 52-weeks, the 12-week NDI scores for those 19 lost to follow-up were carried forward to 52-weeks for purposes of the predictive analysis.

### Baseline analysis

Table 1 presented the mean age, NPRS, and NDI scores between the sexes. None of the differences were significant by independent samples t-tests (p ≥ 0.17) or Mann-Whitney U test (p = 0.88).

### Primary analysis

**Fig 1:**
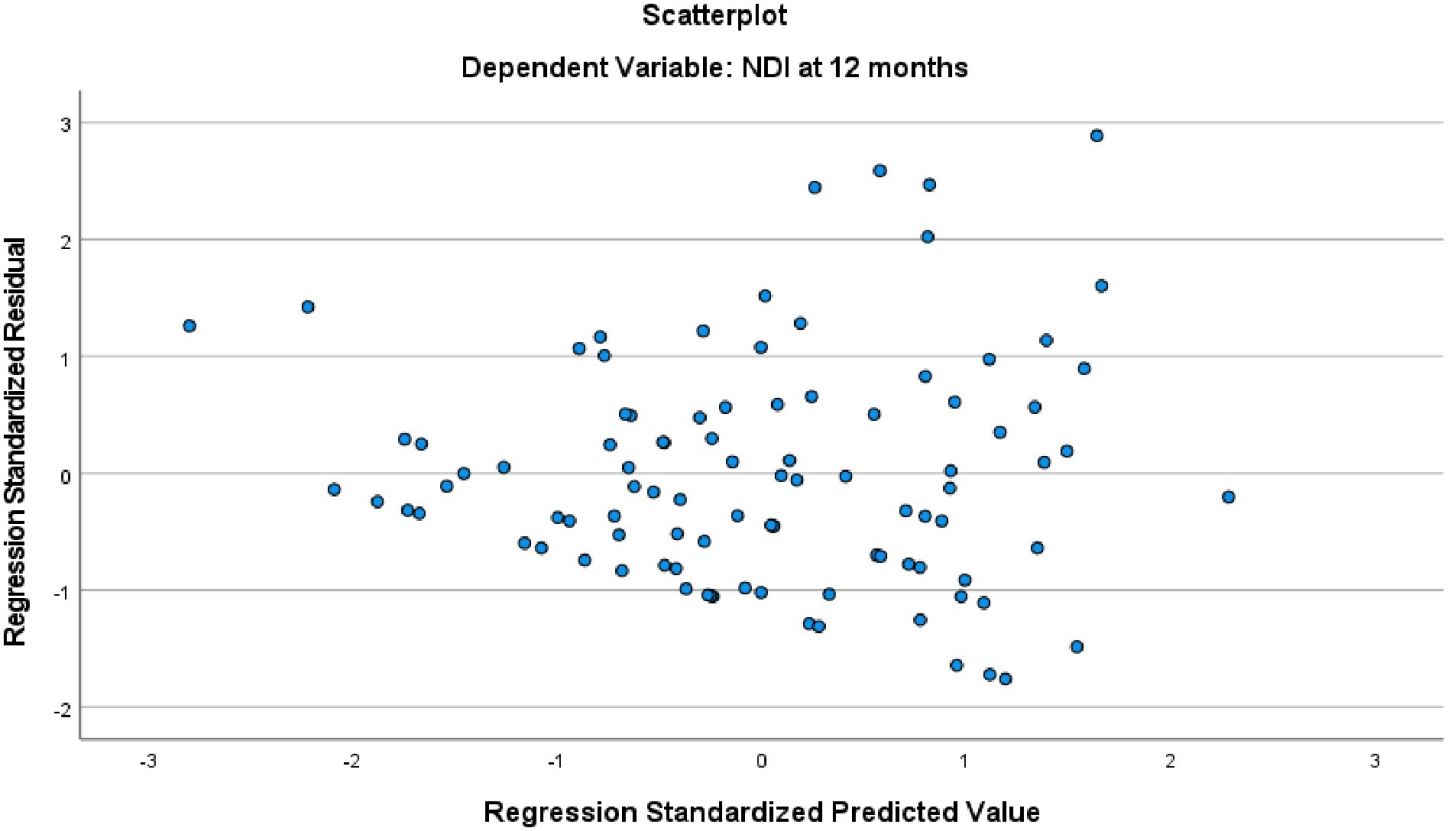
Scedasticity plot of standardized and predicted residuals for the full sample population.

The results from our study demonstrated that the NDI is a more robust tool for males in predicting 52-week disability scores for those with WAD when compared to females, while the NPRS was a more robust tool for females in the same population. Assumptions of linear regression were satisfied, though the scedasticity plot indicated a pattern of increasing residuals proportional to the higher level of NDI scores (Fig 1). Neither age nor sex in isolation explained significant variance in 52-week NDI scores (p of F change ≥ 0.24). In accordance with prior work [4], both baseline NPRS (ΔF = 5.75, p = 0.02, ΔR^2^ = 5.7%) and baseline NDI (ΔF = 9.52, p < 0.01, ΔR^2^ = 8.7%) explained significant unique variance in 52-week NDI scores. In the second model, the sex *x* z-NDI term was non-significant, while the sex *x* z-NPRS term led to a significant increase in variance explained (ΔF = 4.37, p = 0.04, ΔR^2^ = 3.8%). The three significant independent variables explained a cumulative 18.2% of variance in NDI scores captured 52weeks later. Table 2 presents the results of the second regression model including the interaction terms.

**Table 2:** Results of stepwise multiple linear regression using the entire sample.

Variable	β (*β*)	ΔR^2^	ΔF (p)
**Age**	0.10 (0.07)	0.6	0.57 (0.45)
**Sex (male = 1, female = 2)**	1.2 (0.04)	1.5	1.41(0.24)
**NPRS at baseline**	0.39 (0.06)	5.7	5.75 (0.02)
**NDI at baseline**	0.30 (0.34)	8.7	9.52 (<0.01)
**Interaction of sex and NDI baseline**	-2.79 (-0.21)	0.4	0.45 (0.50)
**Interaction of sex and NPRS baseline**	3.31 (0.24)	3.8	4.37 (0.04)

Abbreviations: NPRS, numeric pain rating score; NDI, neck disability index; β: unstandardized beta value; *β*: standardized beta value; p-value significance ≤ 0.05.

The significant interaction term and pattern of non-random residuals supported the decision to conduct the secondary sex-disaggregated regression analyses shown in Table 3. For males, only NDI at baseline was a significant predictor of 52-week NDI score (ΔF = 6.22, p = 0.02, ΔR^2^ = 22.4%). Conversely in females, only NPRS at baseline was a significant predictor of 52-week NDI score (ΔF = 8.28, p < 0.01, ΔR^2^ = 10.5%). Despite the smaller samples in these secondary disaggregated analyses, all assumptions for regression were satisfied after splitting the sample.

**Table 3:** β value (unstandardized and standardized), changes in R^2^, and change in F at each step with significance values for each variable for the regression analyses when disaggregated by sex.

Variable	β (*β*)	ΔR^2^	ΔF (p)
**Males (n = 25)**			
Age	-0.01 (-0.01)	1.9	0.44 (0.52)
NPRS at baseline	-2.19 (-0.30)	0.2	0.04 (0.85)
*NDI at baseline*	*0*.*57 (0*.*62)*	*22*.*4*	*6*.*22 (0*.*02)*
**Females (n = 72)**			
Age	0.12 (0.11)	2.0	1.43 (0.24)
*NPRS at baseline*	*1*.*29 (0*.*21)*	*10*.*5*	*8*.*28 (<0*.*01)*
NDI at baseline	0.20 (0.23)	3.8	3.13 (0.08)

Abbreviations: NPRS, numeric pain rating score; NDI, neck disability index; β, beta value; *p*-value significance ≤ 0.05. *Italicized*: significant predictor variables.

## Discussion

The primary focus of this study was to explore the possibility that common prognostic variables in the field of WAD recovery research exhibit differential functioning by sex of the injured person. The results support this presupposition, that the response to key prognostic variables appear to be different between males and females, and the results of this work should lead to sex-specific prognostic models and screening tools in the interest of more equitable and person-centric research and practice. The significant sex *x* z-NPRS interaction term indicated that a difference in prognostic performance exists, and the secondary sex-disaggregated analyses indicate that initial NDI score may be a more robust prognostic variable in males, while initial NPRS (pain intensity) score may be more robust in females. We are unaware of prior work that has considered moderating or interaction effects in prognostic models in WAD. The results herein indicate that doing so may result in even more accurate prognostic screening tools.

Prior work has identified, for people with WAD, three recovery trajectories people most often follow: rapid/full recovery, delayed/mild persistent problems, and non-recovery/ severe persistent problems [3, 18]. At least one published prognostic screening tool prioritizes baseline NDI score as the first-pass screen for ‘risk of poor outcome’ following WAD [19], though our results indicate that such associations may be considerably more complex than previously considered. Two such findings are worth exploration in this regard: the first is the scedasticity plot in Fig 1, the second are the significant sex interaction term and sex-disaggregated analyses. These will be considered in turn.

The pattern of residuals in Fig 1 suggests the predictive value of the regression model is better when 52-week NDI scores are on the lower end of the scale, and less precise as 52-week scores increase. These findings fit with a recent publication using quantile regression on a dataset of which the current sample was a subset but using different predictor variables [18]. In that study, Modarresi and colleagues similarly found the prognostic accuracy of many commonly reported variables (post-traumatic distress) was better when recovery outcomes were good, but less accurate when recovery outcomes were poor. In practice, such results make analysis and interpretation of any such work in the field more complex, but for injured people these offer promise–while those deemed ‘low risk’ at baseline are indeed likely to recover quickly, those deemed ‘higher risk’ are part of a group with considerable variation in 52-week outcomes, some of which is presumably modifiable. From a screening and social justice perspective this is also valuable–most current clinical practice guidelines endorse minimal intervention for acutely injured people deemed low risk, so accuracy of such judgements is of critical importance at the lower ends of the scale. Conversely, those deemed higher risk usually move on to more fulsome clinical workup, meaning that even when risk screening is inaccurate the salient impact on the injured person is undergoing more evaluation.

The differential prognostic functioning by sex demands further exploration and replication in other samples, especially considering the smaller sample sizes in the disaggregated analyses. If replicated, such a finding will demand a rethinking in how prognostic screening tools and decision rules are created. The findings herein may go some way towards explaining the inconsistencies in prior work around sex itself as a prognostic variable. For example, a meta-analysis by Walton and colleagues [10] found that females showed a small but significant 1.5-fold increased odds of non-recovery after WAD, but work since then has rarely found sex to be an important prognostic discriminator [20–22]. Perhaps, per the findings of this study, it is not sex itself that is the prognostic variable, but rather the effect that sex has on other variables that has led some groups to find it significant while others have not. If replicated, the simple interpretation of such findings is that prognostic tools should be created and interpreted separately for males and females, an endorsement that has been supported by the SAGER group and several other critical feminist and disability scholars [23–25]. The mechanisms to explain why pain intensity appears to be more relevant for females while pain-related disability is more relevant for males is a novel finding for which we are unable to find supporting prior evidence, suggesting this is another area that demands further quantitative and qualitative exploration.

We acknowledge an important drawback to the interpretations of this work that are borne by the level of data collected in the original parent study. We have consistently used the term ‘sex’ throughout this study, as the initial intake data form offered respondents binary response options of selecting male or female. This is most closely aligned with the biological concept of sex-assigned-at-birth, referring to biological differences such as reproductive organs and circulating hormones. However, it is now well-recognized that sex, as a biological construct, should be considered distinct from the concept of gender, including socially or culturally constructed traits, roles, and behaviors. Gender is a difficult construct to quantify in post-positivist research, though some tools exist and may be useful for components of such research [26]. Members of our group have recently found that constructs of relationship-orientation and emotiveness, commonly ascribed to more feminine-leaning traits by Western cultural standards, were more strongly associated with acute pain and post-traumatic distress in an independent sample analysis using the Gender, Pain, and Expectations Scale [manuscript currently under review]. So, while the results reported herein appear to indicate that it is sex, rather than gender, that requires differential consideration in prognostic studies, we need to make clear this is a side-effect of the data available and instead suggest these concepts require considerable further exploration to better determine what are the important constructs, and how biological sex and cultural gender norms may interact to better predict outcomes from WAD.

Finally, we support the SAGER guidelines in endorsing sex- and gender-based analyses not only in prognostic research described here, but also in intervention trials. It is widely-recognized the evidence base to support many interventions for WAD, whether acute or chronic, is weak and/or demonstrates generally small clinical effects [27, 28]. Based on our recent results including those described here, we propose one explanation for limited empirical support for one intervention over another is that prior work in the field has rarely been designed with sex-disaggregated sub-analyses in mind. It seems possible, and very reasonable, to believe the effects of interventions, be they pharmaceutical, rehabilitative, surgical, or otherwise, may vary by sex. This seems like a ripe area for further exploration.

## Limitations

The primary limitation in this study was the unequal numbers of males (25) and females (72). While not equal, the nearly 1:3 ratio is reflective of the proportions commonly reported in research studies and clinical practice [14, 29, 30], and preparatory analyses of between-group variance indicated similar variances despite the unequal sizes. As a secondary analysis of previously collected data we were unable to recruit more acutely injured male participants, and as such these analyses should be considered exploratory and hypothesis generating at this stage. Future studies should aim to have equal numbers between the sexes.

A second limitation was the loss of 19 participants between 12 and 52 weeks. Although we felt comfortable carrying NDI and NPRS scores forward from the 12 to the 52-week time point, as this allowed data from all 97 people to be included in the analysis using an intention-to-treat approach, we recognize it is unlikely that all followed the same trajectory during that time. Our decision was based on previous research showing minimal change in these scores between 12 and 52 weeks [3].

A final limitation is the examination of just three primary variables (age, baseline NDI scores, and baseline NPRS). The purpose of this study was not to evaluate all potential variables; thus, we used these three established variables [4, 21] as a proof-of-concept to explore possible sex-related differences and to better inform future research designs in the area. While there are numerous variables associated with the development of chronic WAD such as hyperarousal scores on the post-traumatic diagnostic scale [21], increased levels of muscle fatty infiltrate within the cervical musculature [31], or levels of post-traumatic stress [32]; evaluating how males and females react to each one should be included in future research designs [11].

## Summary

The results from this study reproduce prior findings of an association between NDI scores when captured 52-weeks apart [33]. The data provide at least preliminary evidence that males and females may have different prognostic variables associated with the development of chronic WAD following an MVC, with baseline NDI scores predictive for males and baseline NPRS for females. Such information could potentially assist in the development of and a greater understanding for the prognostic profiles of people following an MVC with a target to explore, test, and establish more efficient treatment strategies.

## Supporting information

S1 Checklist STROBE statement—Checklist of items that should be included in reports of *cohort studies*. (DOCX)
